# pH responsive cationic guar gum-borate self-healing hydrogels for muco-adhesion

**DOI:** 10.1080/14686996.2023.2175586

**Published:** 2023-02-28

**Authors:** Athira Sreedevi Madhavikutty, Arvind K. Singh Chandel, Ching-Cheng Tsai, Natsuko F. Inagaki, Seiichi Ohta, Taichi Ito

**Affiliations:** a Department of Chemical System Engineering, The University of Tokyo, Tokyo 113-8656, Japan; b Center for Disease Biology and Integrative Medicine, The University of Tokyo, Tokyo 113-0033, Japan; c Department of Bioengineering, The University of Tokyo, Tokyo 113-8656, Japan

**Keywords:** Self-healing, hydrogels, muco-adhesion

## Abstract

We developed a new muco-adhesive hydrogel composed of cationic guar gum (CGG) and boric acid (BA). The CGG-BA precursor solution of 0.5–2% w/v concentration exhibited fluidity at low pH (3–5), while gelation occurred within 1 min at physiological pH (7–8) conditions. Scanning electron microscopy and Fourier-transform infrared spectroscopy results confirmed the change in physical and chemical behavior, respectively, with change in pH. The pH-responsive self-healing ability was analyzed through microscopy and rheology. CGG-BA hydrogels showed good self-healing property at pH 7.4. The *in vitro* biocompatibility test of the hydrogel studied using NIH3T3 and NHEK cells showed that it was non-toxic at concentrations of CGG-BA below 2% w/v. *Ex vivo* mucoadhesive tests confirmed the hydrogel’s potential for use as a muco-adhesive. Burst pressure tests were conducted using pig esophageal mucosa and the results showed that at pH 7.4, 1% w/v CGG-BA self-healable hydrogel resisted about 8 ± 2 kPa pressure, comparable to that of Fibrin glue. This was higher than that at solution (pH 5) and brittle gel (pH 10) conditions. To confirm the good adhesive strength of the self-healable hydrogels, lap shear tests conducted, resulted in adhesive strengths measured in the range of 1.0 ± 0.5–2.0 ± 0.6 kPa, which was also comparable to fibrin glue control 1.8 ± 0.6 kPa. Hydrogel weight measurements showed that 40–80% gel lasted under physiological conditions for 10 h. The results suggest that CGG-BA hydrogel has potential as a pH responsive mucosal protectant biomaterial.

## GRAPHICAL ABSTRACT

**Figure:**
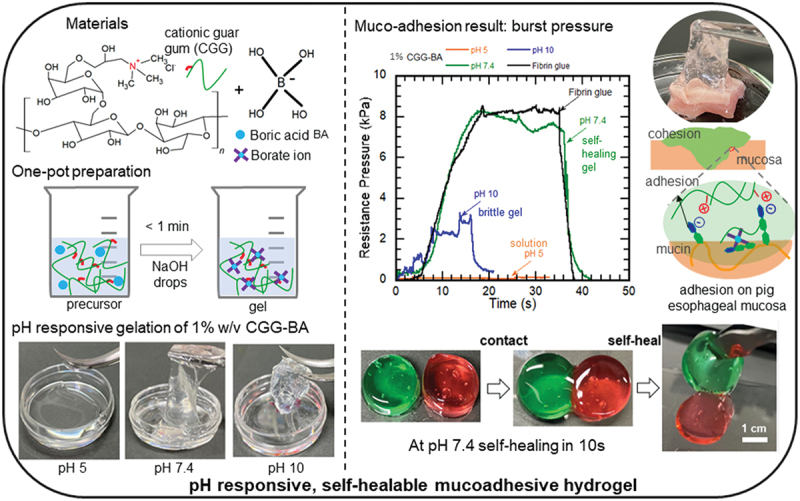

## 1. Introduction

Mucoadhesive materials [1,2] are becoming crucial in wound healing and drug delivery systems, a vital part of minimally invasive therapies such as endoscopy. Polymeric systems [3,4] in combined, substituted, or conjugated forms have been developed for sustained muco-adhesion [5–7]. For, e.g., hydrogels developed from polymers provide sufficient retention time in the oral cavity, adequate drug penetration, as well as high efficacy and patient acceptability [8]. Mucosa is the protective layer covering various cavities such as oral and nasal cavity, esophagus, stomach, intestine, and eyes [9]. They consist of a glycosylated protein layer called mucin. The gastro-intestinal mucosal conditions vary from highly acidic (pH 1.5–2.5) in gastric mucosa, to neutral (pH 7) in esophageal mucosa, to slightly alkaline (pH 7.5 ± 0.4) in intestinal mucosa [10]. pH of the eye mucosa [11] is almost neutral between 7 and 7.3. The nasal mucosal pH increases from 5.5–6.5 at normal conditions to 7.2–8.3 during rhinitis (inflammation) [12]. Due to the difference of pH at different mucous tissues, pH responsive mucoadhesive polymers and hydrogels would be able to adhere to specific mucin layer and protect it. Though many muco-adhesives are available [13], there is a rising need for new materials that are biocompatible, easily prepared, and cost-effective.

Cationic polysaccharide-based hydrogels such as chitosan are mucoadhesive owing to their interactions with negatively charged mucin layer of mucosa [14]. Cationic, quaternary ammonium hydroxyl-propyl modified guar gum (CGG) [15] is a commercially available, low-cost galactomannan polysaccharide [16–19] utilized in food, cosmetics, water treatment, paper industry, etc. CGG is more soluble in water compared to pure guar gum due to the presence of the quaternary groups [20,21]. CGG-based hydrogels were developed for biosensor application due to its stretchable and self-healing properties [22]. However, except few studies [23], CGG was not extensively analyzed for its potential as a mucoadhesive biomaterial. CGG is expected to have muco-adhesive potential due to its biocompatibility [19] and the presence of cationic charge within its structure.

Polymer-based hydrogels [24] can interact with tissues, making them potential mucoadhesive biomaterials. Particularly, self-healing hydrogels [25], owing to the dynamic crosslinking are being explored for durable muco-adhesion. Previous studies [26–28] showed that materials with boronic acid functional groups are a novel class of muco-adhesives. However, such materials require inert conditions at high temperatures for long hours for their preparation. Therefore, new alternative hydrogels with simple and quick preparation methods are required [29]. In this regard, boric acid crosslinked hydrogels are potential muco-adhesives. Boric acid at moderate amounts has been used in food additives [30] and eye drops [31]. When used at suitable concentrations, it can be useful as a mucoadhesive. Natural polysaccharides such as guar gum and synthetic polymers such as poly-vinyl alcohol (PVA) that contain two or more diol units in their structure are known to form hydrogels via the ‘di-diol’ complexation with borate ions [32–34]. These borate-based hydrogels have been found to be flexible and exhibit self-healing behavior [35].

In this study, we newly developed a quick gelling (less than 1 min), pH responsive, mucoadhesive hydrogel composed of CGG and boric acid (BA) suitable for use in physiological neutral and alkaline environments. For hydrogel preparation, we used a simple one-pot synthesis technique. The change of rheological characteristics of CGG-BA with pH was analyzed by frequency sweep, strain sweep, and yield stress measurements. The tackiness effect of pH was also analyzed. *In vitro* biocompatibility was evaluated using NIH3T3 and NHEK cells. The self-healing behavior of the hydrogels was observed and measured using rheological measurements. We compared the properties of the CGG-BA solution, self-healing hydrogel, and brittle hydrogel for its suitability as a mucoadhesive. Mucoadhesive function was studied using *ex vivo* methods, viz., burst pressure, tackiness, lap shear, and gel weight test.

## 2. Experimental section

### 2.1. Materials

Cationic guar gum (Jaguar® Excel; Mw = 1,000,000 − 1,500,000, degree of substitution = 0.10 − 0.13) [15] was kindly provided by Sansho Corporation (Tokyo, Japan). Boric acid (H_3_BO_3_), sodium hydroxide (NaOH), potassium chloride (KCl), sodium phosphate dibasic, potassium phosphate monobasic, sodium chloride (NaCl), Dulbecco’s modified Eagle’s medium (DMEM), penicillin-streptomycin-amphotericin B suspension (PSA-B), and fetal bovine serum (FBS) were purchased from Fujifilm Wako Pure Chemical Industries Ltd. (Osaka, Japan). Fibrin glue, Beriplast® P, was obtained from CSL Behring (Pennsylvania, United States).

### 2.2. Preparation of CGG-borate hydrogel and determination of gelation time

**Figure 1.**
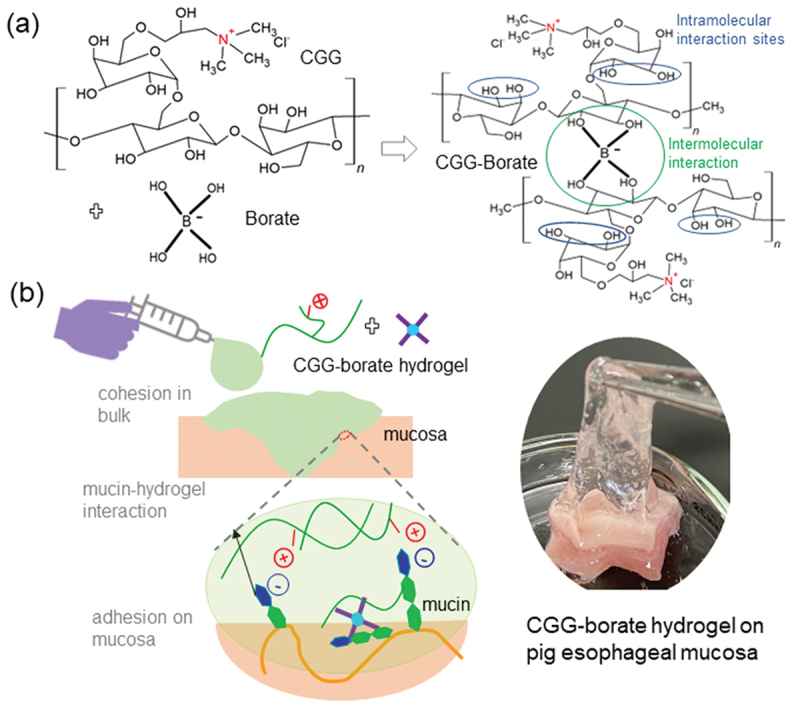
(a) Crosslinking reaction between CGG and BA, (b) Expected muco-adhesive potential of CGG-BA hydrogel.

As suggested by previously reports [33,34], BA forms both intermolecular and intramolecular complexes with diol groups of galactomannans. Here, the interactions depend on the number of diol groups in CGG and BA molecule. Considering both complexations as shown in Figure 1, the amount of CGG and BA to be combined was such that the mole ratio of CGG to BA was 1.0 to 1.5 (equivalent to mass ratio of 7.19 to 1). Pre-weighed quantities of CGG were taken in a glass beaker, and then normal saline (0.9% w/v of NaCl) was added and CGG solution was prepared by stirring at 1000 rpm for 2–4 h. Equal volumes of precalculated quantities of CGG and BA were mixed while stirring at 250–500 rpm to form 1%, 2%, 3%, and 4% w/v CGG-BA precursor solution. The resulting solutions had pH ranges between 5.0 and 7.4. Then, 10 mM HCl or 10 mM NaOH was added dropwise to adjust the pH to 5, 6, 7, 7.4, 8, and 10.

The pH-responsive gelation time of the hydrogels was measured using the stirring method previously reported [36]. Here, we first add 100 µl of the CGG-BA precursor solution to a stirrer bar of size 2 × 5 mm. Then, 5 µl volume of 10–100 mM NaOH was added to the precursor stirring at 200 rpm to adjust pH. During mixing, the gelation was observed, and the gelation time was noted as the time at which a hydrogel globule was formed.

As 1% w/v CGG-BA precursor could be passed through a 2.1 mm diameter endoscopic tube of length 1.65 m by hand injection, this concentration was used as standard in all ensuing experiments unless specified [37].

### 2.3. Characterization of formed hydrogel

#### 2.3.1. Rheological analysis: frequency sweep, strain sweep, and alternate strain

Rheological measurements were conducted using a rheometer (MCR 302, Anton Paar, Graz, Austria). For frequency sweep, strain sweep, and alternate strain measurements, hydrogel disks of 25 mm diameter and 1 mm height were prepared. 1% w/v CGG-BA gels at different pH values were subjected to frequency sweep, amplitude sweep, and alternate strain. We also studied the effect of concentration (0.5, 1, 1.5, 2% w/v) of the hydrogel. Frequency (*ω*) sweep conducted between a range of 1–100 rad s^−1^ measured the variation in storage moduli (*G′*) and loss moduli (*G″*). Amplitude sweep measured the strain (1–1000%) dependency on *G′* and *G″* that in turn calculated the stress dependency and yield stress of the hydrogels. To study the self-healing property, rheological analysis of hydrogel-restructuring was done by application of alternate amplitude sweep (five cycles each of 120 s) at low 1% and high strains 1000% strains at a fixed frequency (*ω* = 6.28 rad s^−1^) [38,39]. In the above experiments, to avoid hydrogel slippage, we used a sandpaper on the bottom plate [40].

We conducted a tackiness test using the rheometer probe and plate setup to analyze the effect of pH on the stickiness and cohesiveness of the hydrogel [41]. For this, hydrogels of 25 mm diameter and 0.25 mm thickness were placed on the plate. The probe was adjusted to touch the hydrogel at the 0.25 mm gap for 5 min to achieve an equilibrium. The probe was then quickly moved up axially at a speed of 5000 µm s^−1^ (300 mm min^−1^), and the corresponding contact force was recorded. The maximum force at which debonding occurred was determined as tack force. All of the above experiments were done in triplicate.

#### 2.3.2. Scanning electron microscopy (SEM) analysis

1% w/v CGG-BA hydrogels at pH 5, 6, 7, 7.4, 8, and 10 were lyophilized for 48 h, prior to the SEM analysis. The surface morphology of lyophilized hydrogels at different pH values were examined using SEM (Mini scope® TM3030 Plus, Hitachi, Tokyo, Japan) at 5 kV [23].

#### 2.3.3. Fourier-transform infrared (FTIR) spectroscopy analysis

Lyophilized hydrogels mentioned in Section 2.3.2 were also used for FTIR measurements. To study the effect of pH on the structural interactions of CGG and BA, the absorbance spectra between wavelengths of 4000 cm^−1^ and 400 cm^−1^ were recorded using a FTIR spectrometer (4200ST, JASCO, Tokyo, Japan) using potassium bromide disk method [21]. Spectragryph software was used to analyze and compare the obtained results.

#### 2.3.4. Analysis of self-healing behavior

The self-healing behavior of hydrogels was analyzed by visual observation of alternate bonding and re-bonding. The hydrogels were colored using red and green food grade coloring pigments (Kyoritsu Foods Co., Ltd.) and molded into disks each of 1.5 cm diameter and 1 cm thickness. At first, the hydrogel disks were brought into physical contact. If the hydrogel disks adhered together, the point of contact between them was cut using a blade and the separated disks were brought together again [42]. The rheological analysis of self-healing was mentioned in Section 2.3.1. With reference to previous studies [43,44], to analyze self-healing at a microscopic scale, the hydrogel cut with a blade was observed through a confocal microscope (FluoView FV 3000, OLYMPUS, Tokyo, Japan). The images were taken at intervals of 10 s, 1 min, 5 min, 1 h, and 5 h.

### 2.4. Cytotoxicity of CGG-borate hydrogel

The cytotoxicity of the CGG-BA hydrogels was evaluated based on the WST assay using a mouse fibroblast cell line (NIH3T3, RIKEN Cell Bank) and a normal human epidermal keratinocytes (NHEK) cell line. NIH3T3 cells were cultured in high glucose DMEM with 10% FBS and 1% PSA-B and the NHEK was cultured in a keratinocyte growth medium-2 with 1% PSA-B, at 37°C and 5% CO_2_ condition. After reaching 70%–80% confluency, the cells were harvested with 0.25% trypsin solution, seeded on a 24 well plate at a density of 3 × 10^4^ cells per well and then incubated at 37°C and 5% CO_2_ overnight. The medium was then replaced with a medium containing material sample with various concentrations, followed by the further incubation for 48 h (*n* = 4). The cells were then observed through confocal microscopy and images were taken. The WST assay was performed by adding a WST reagent and subsequent incubation for 90 min. A plate reader was used to measure the absorbance at 465 nm (2030 ARVO V3; PerkinElmer, Waltham, Massachusetts, USA). The cell viability was calculated by normalizing absorbance values to those of wells where no test materials were added to the media using Equation (1).

(1) $$Cell\;viability=\frac{Abs_{sample}-Abs_{blank}}{Abs_{control}-Abs_{blank}}$$

where Abs_sample_, Abs_control_, and Abs_blank_ are the absorbance of the sample (materials added), control (cells in a medium without material), and blank (only medium), respectively [45].

### 2.5. Ex vivo muco-adhesion of CGG-borate hydrogel

#### 2.5.1. Burst pressure test

**Figure 2.**
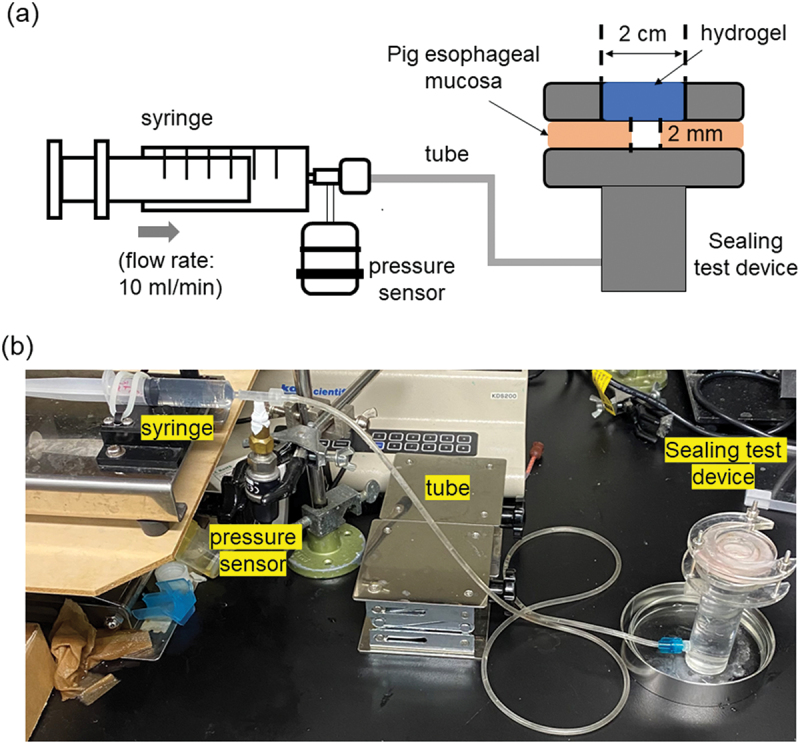
(a) Schematic of the burst pressure measurement, (b) Image of the experimental setup.

Further, to study the sealing effect of hydrogels, an *ex vivo* burst pressure experiment [46] was conducted. The experimental set up was assembled by referring to a previously published [47] method with few modifications. A piece of esophageal mucosa of diameter 5 cm was cut out from pig esophagus obtained from Tokyo Shibaura Organ Co., Ltd., then placed tightly over the custom-made sealing device (Figure 2). The device was assembled such that a circular gap of diameter 2 cm exposed the mucosal tissue. A 2 mm diameter incision was made on top of the tissue using a biopsy punch. A total of 1 ml each of 1% w/v CGG-BA at pH 5, 7.4, and 10 were placed in the gap over the mucosa, covering the incision. Fibrin glue was also tested as a control material. The thickness of the hydrogels applied was roughly 4 mm. All the samples were incubated at 37°C for 30 min before measurement. The resistive pressure that the hydrogel can withstand when phosphate-buffered saline (PBS) was ejected at a flow rate of 10 ml min^−1^. The flow was controlled by a syringe connected to a syringe pump (ELCM2WF 10K-AP, Oriental Motor, Tokyo, Japan) [48]. The pressure drop resisted by the hydrogel over the tissue was measured using a pressure gauge (AP-12s, Keyence Corporation, Osaka, Japan). The pressure-drop corresponding to the failure of the gel was noted as the burst pressure.

#### 2.5.2. Lap shear test

A lap shear test was conducted to determine the hydrogels adhesive strength on porcine esophageal mucosa (same as previous section) through a 180° peeling test. The porcine mucosa was cut into a uniform size of 3 cm × 1 cm (length × width). The hydrogel was applied to the surface of the mucosa such that the contact surface was 1 cm × 1 cm. The substrate was then placed between the probes of a tensile rheometer (CR-100, Sun Scientific. Co. Ltd., Tokyo, Japan). The substrate was pulled apart at a speed of 10 mm min^−1^ and the corresponding force was recorded. The adhesive strength is calculated by Equation (2)

(2) $$Adhesive\;strength\left(Pa\right)=\frac{Max.\;Contact\;Force\;\left(N\right)}{Contact\;area\;\left(m^{2}\right)}$$

#### 2.5.3. Degradation test

Gel weight degradation method was used to determine the stability of the adhered hydrogels on the mucosal surface under physiological conditions [49,50]. We applied about 500 mg of hydrogels (1%, 1.5%, and 2.0% w/v at pH 7.4) on the mucosa and placed the tissue in PBS (pH 7.4), followed by the incubation at 37°C. The weight of the hydrogel was measured over time (*t*) at certain intervals and then the percentage of detached hydrogel is determined by Equation (3)

(3) $$\%\;dettachment=\frac{Weight\;at\;time\;t-Initial\;weight\;at\;t=0}{Initial\;weight\;at\;t=0}\times 100$$

## 3. Results and discussion

### 3.1. pH dependent gelation

**Figure 3.**
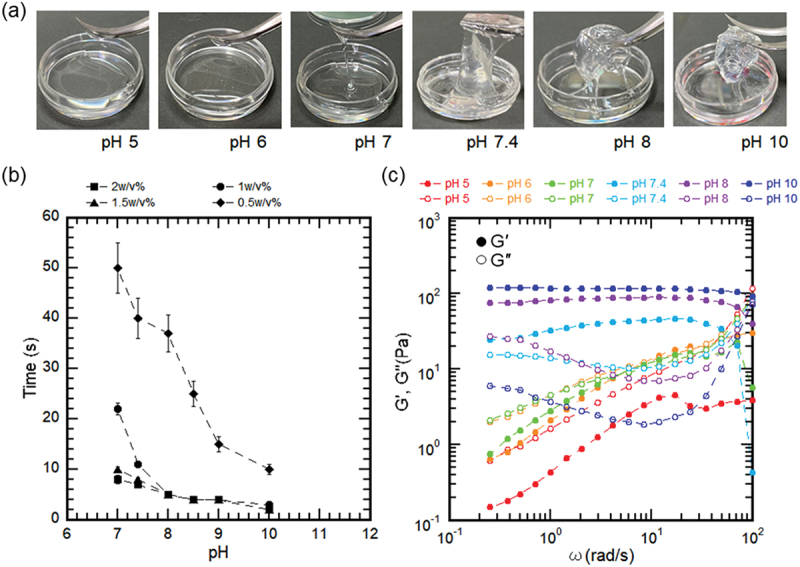
Effect of pH (5, 6, 7, 7.4, 8, 10) on CGG-BA mixtures. (a) Images of CGG-BA mixtures, (b) Gelation time of CGG-BA mixtures, (c) Frequency sweep of CGG-BA mixtures.

Figure 3(a) shows images of change in the appearance of 1% w/v CGG-BA with different pH, from 5 to 10. At pH 5 and 6, no gelation was observed, and the sample remained in a solution state. By increasing the pH to 7, hydrogel formation became visible. When pH was about 7.4, the hydrogel showed a slimy texture. Further increase of pH beyond 8 showed the formation of a brittle hydrogel.

The gelation time decreased with an increase in pH. At pH less than 7, the precursor remained in the solution state, hence the gelation time could not be determined. As shown in Figure 3(b), at 1% w/v CGG-BA concentration, an increase in pH from 7 to 10 decreased the gelation time from 22 ± 1s to 2s. A representative image and illustration of gelation determination are shown in Figure S1(a – b). At pH 7, when concentration increased from 0.5% to 2% w/v, the gelation time decreased from 50 ± 6s to 8s. The results suggest that the material has the potential for pH responsive *in situ* gelation.

The frequency sweep dependencies of storage modulus (*G′*) and loss modulus (G″) of 1% w/v CGG-BA hydrogel at different pH are shown in Figure 3(c). The value of the storage modulus *G′* increased from 1 Pa at pH 5 to 100 Pa at pH 10. At pH 5, 6, and 7, the relationship between *G′* and *G″* was *G′* < *G″*, while at pH 7.4, 8, and 10 it was *G′* > *G″*. A plot of loss factor (tanδ = *G″*/*G′*) as a function of pH showed tanδ > 1 at pH 6 and 7, whereas at pH > 7, it showed tanδ < 1 (Figure S1(c)). These results suggested that a transition from solution to hydrogel occurred around physiological pH.

### 3.2. SEM images

**Figure 4.**
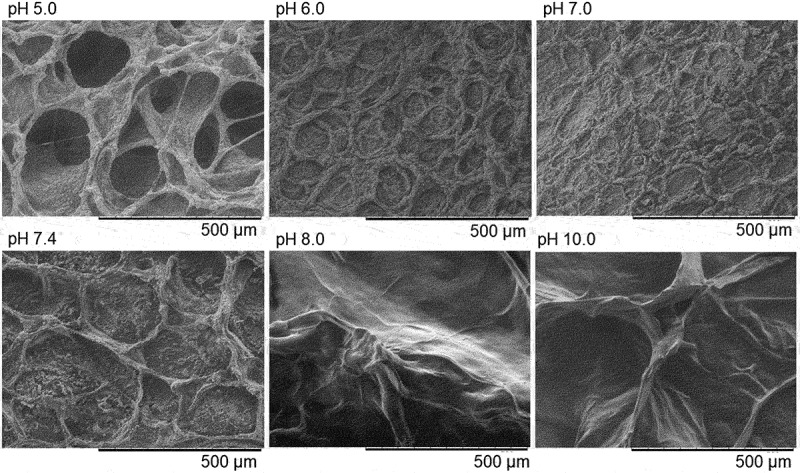
SEM images (resolution 500μm) of CGG-BA with different pH.

SEM images of CGG-BA of resolution 500 μm at different pH values are shown in Figure 4. The CGG-BA system at pH 5 showed morphology similar to that of uncross-linked CGG polymers as shown in Figure S2(a). However, unlike images of CGG, CGG-BA at pH 5, showed particles of undissociated BA, visible in higher resolution (100 μm) images (Figure S2(b)). The undissociated BA remained till pH reached about 8. At low pH, BA remained in the undissociated state (dissociation constant of boric acid = 8.5) [51], whereas at high pH, it dissociated to form borate ions that cross-linked with CGG. The increase in pH showed a transition from polymer-like state at pH 5 to a more stretched state above pH 7. This is due to the presence of a borate cross-linking within the system [52]. We note that since the hydrogels were freeze-dried for these SEM observations, the obtained images do not represent the exact structure of the hydrogels at the original state.

### 3.3. FTIR analysis of interactions between CGG and BA at varying pH

**Figure 5.**
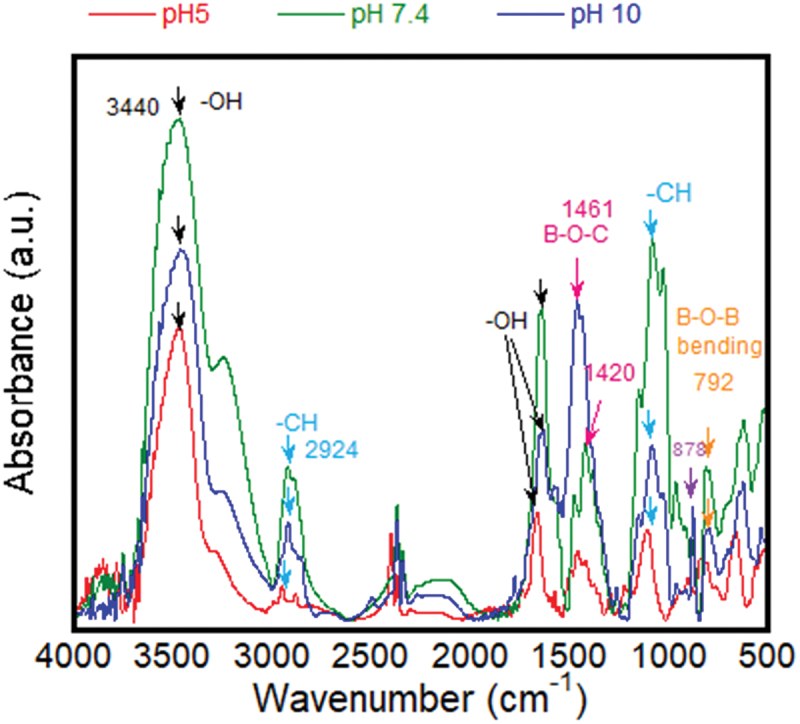
FTIR spectra of CGG-BA with pH = 5, 7.4, and 10. Arrows indicate the characteristic peaks.

To study the observed effect of pH on the physical nature of the hydrogels, the pH dependence on chemical cross-linking between CGG and BA was measured using FTIR. Figure 5 shows the FTIR spectra of 1% w/v CGG-BA at pH 5, 7.4 and 10. There is an increase in the peak of -OH (at 3440 and 1520 cm^−1^) for pH 7.4 compared with pH5 and pH10. This was due to an increased hydrogen bonding within the system. At pH 5, the hydrogen bonds would be too weak, and there are no -OH peaks of borate ions as BA remained in the undissociated form. With an increase in pH from 7.4 to 10, there was a shift in the B-O-C peak from 1420 to 1461 cm^−1^. This would be due to the strong complexation of borate and CGG at pH 10 compared with the weak interactions at pH 7.4. In addition, at pH 10, there is a strong peak at 878 cm^−1^ due to the presence of residual borate ions that are not cross-linked with CGG. This peak was absent at pH 5 and 7.4. The results complied with previously reported data [21]. Also, the interactions within the CGG-BA system are like those of other polysaccharide-BA materials [53–55]. Figure S3 shows a schematic representation of the interactions between CGG and BA at pH 5, 7.4 and 10.

### 3.4. Yield stress and tackiness of hydrogels

**Figure 6.**
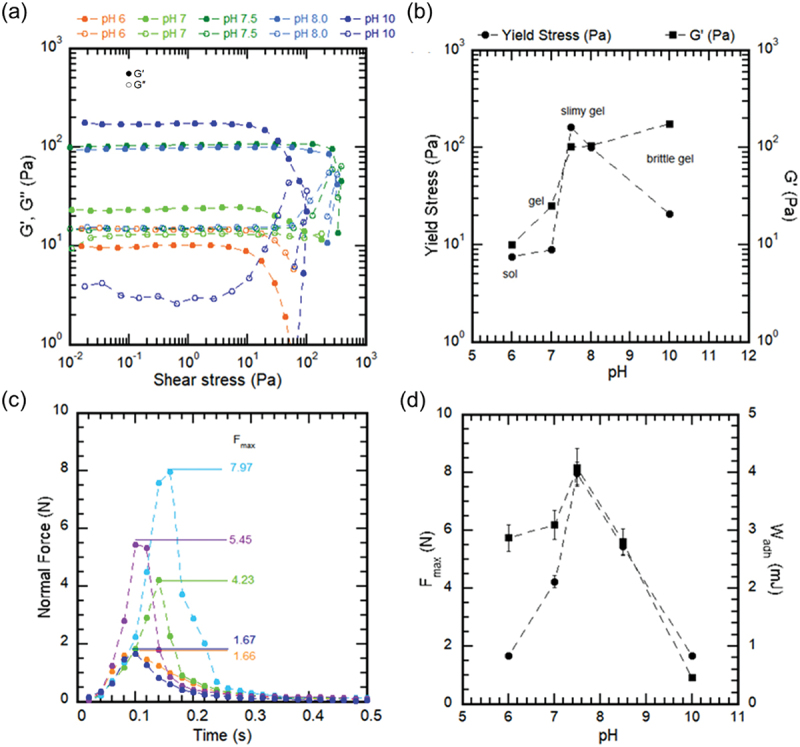
Effect of pH on yield stress and tackiness of CGG-BA hydrogels. (a) *G′* and *G″* as a function of shear stress on the hydrogel, (b) *τ*_*y*_ of hydrogels at different pH. (c) Normal force exerted on the hydrogel during the tack measurement as a function of time, (d) the tackiness force (*F*_max_) and work of adhesion (*W*_adh_) of hydrogels at different pH.

Yield stress (*τ*_*y*_) of 1% w/v CGG-BA hydrogel at different pH values, and the results are shown in Figure 6(a). For the solution at pH 5, there was no measurable *τ*_*y*_. *τ*_*y*_ increased with an increase in pH. Value of *τ*_*y*_ increased from 10 Pa at pH 6, reached a maximum of 200 Pa at pH 7.4, then decreased to 20 Pa at pH 10. As the yield stress property prevents material flow from the applied surface, an intermediate pH of 7.4 would be suitable for application to tissues. At pH 5, the resistance to flow was lowest at pH 5 due to the solution-like nature of CGG-BA. At pH 10, the hydrogel existed in a brittle state, and broke quickly upon application of high stress that resulted in low *τ*_*y*_. It is to be noted that, like the frequency sweep results, the value of storage modulus (G’) increased with pH (Figure 6(b)). This would be due to an increase in the stiffness of the hydrogel due to an increase in borate cross-linking. The ability to withstand stress also depended on hydrogen bonding [56,57].

As shown in Figure 6(c), when the probe is pulled away from the lower plate of the rheometer, the resistance force increases, reaches a maximum, and then decreases as debonding occurs. The maximum normal force (Figure 6(d)) was 1.7 ± 0.2 N at pH 5, increased to about 8.0 ± 0.5 N at pH 7.4, and decreased to 1.7 ± 0.3 N at pH 10. The work of adhesion (W_adh_) is the area under the force vs. time curve. W_adh_ was calculated and obtained via the rheometer software. Higher tackiness implied that the hydrogel had a higher tendency to stick to surfaces and to itself. This was evident from the images of the hydrogels stuck to the rheometer plates during their movement upward (Figure S4). Tackiness is widely used in the food industry to categorize diverse food substances by evaluating their sticky nature. A material is considered tacky when it possesses the right balance between softness and the ability to dissipate energy [41]. However, our experiment has the drawback that it cannot provide insights on the mechanism of adhesion as it does not consider the possible substrates to which adhesion can take place. Here, the focus was simply to analyze the effect of pH on cohesion within the hydrogel network and the results corresponded to that of yield stress measurements.

The yield stress and tackiness behavior showed similar trends due to the dependence on the nature of the cross-linking within the hydrogel as shown by the FTIR analysis. At pH 5, as the BA remained undissociated, no interaction occurred between the CGG and BA. Therefore, there was no yield stress or tackiness. As the pH is increased, dissociation of BA to form borate ions occurs. At this condition, the borate ion forms two types of bonds with CGG: hydrogen bonds with the -OH groups and the di-diol cross-linking. At the intermediate pH range, e.g. pH 7.4, both hydrogen bonding and borate cross-linking were responsible for binding the system together, allowing them to withstand high stresses. On the other hand, at high pH, i.e. pH 10, all the borate ions complexed to CGG, decreasing the hydrogen bonding of the system. This led to a more hydrophobic and brittle network that was non-sticky.

### 3.5. Self-healing behavior of CGG-BA hydrogel

**Figure 7.**
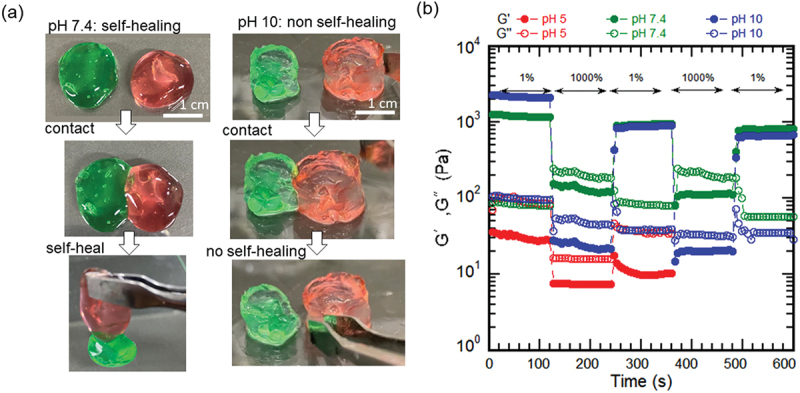
Self-healing behavior of CGG-BA at different pH. (a) Images of 1% CGG-BA when two cut pieces of hydrogel with pH 7.4 and pH 10 were brought in contact. Scale: 1 cm, (b) Rheological evaluation of restructuring of 2% w/v hydrogels at pH 5, 7.4, and 10 by subjecting to alternate low (1%) and high (1000%) strains.

The self-healing behavior of CGG-BA hydrogels was examined using previously reported methods [58]. Two hydrogel disks were brought together to contact. Then were cut and separated, and then brought back to get the cut surface contacted. Upon recontact, the hydrogels at pH 7.4 showed almost immediate self-healing (a few seconds) as shown in Figure 7(a). The observed immediate self-healing would be due to the quick reformation of bonds. However, at pH 10, the gels did not re-bond when brought in contact (Figure 7(b)). The behavior was the same for hydrogel concentrations of 1%, 1.5%, and 2% w/v as shown in Figure S5(a). Supporting Information file 2 (.MOV) shows a representative video of the self-healing of 2% hydrogel at pH 7.4. The alternate high and low strain cycles of CGG-BA hydrogels using rheometers also confirmed the self-healing behavior due to restructuring of broken bonds Figures 7(c) and S5(b). More images of CGG-BA hydrogel’s ability to withstand stress by pulling after self-healing are shown in Figure S5(c).

To visualize the self-healing on a microscale [43,44], a small tear was made on the 1.0% w/v hydrogel with pH 7.4 placed on a glass plate and observed under the microscope. The self-healing of the hydrogel over time was evident by the microscopic images (Figure S6) of the cut made on the self-healing hydrogel. The cut made on the hydrogel was found to heal over time. The healing was expected to initiate within 10 s of microscope adjustment. Within a minute, the hydrogel had fully healed. The state remained the same for about 5 h of observation.

The rapid self-healing of the CGG-BA hydrogel could be due to the presence of hydrogen bond between the borate group and diol groups of CGG. The rapid association and dissociation of hydrogen bonds has been reported to occur on time scales of picosecond [35]. The hydrogen bonding within the CGG-BA hydrogel can be attributed to the intramolecular bonds of diol groups of CGG and the intermolecular bonds between CGG and borate. The proposed self-healing mechanism is represented as a schematic in Figure S7.

### 3.6. Cytotoxicity of hydrogels

**Figure 8.**
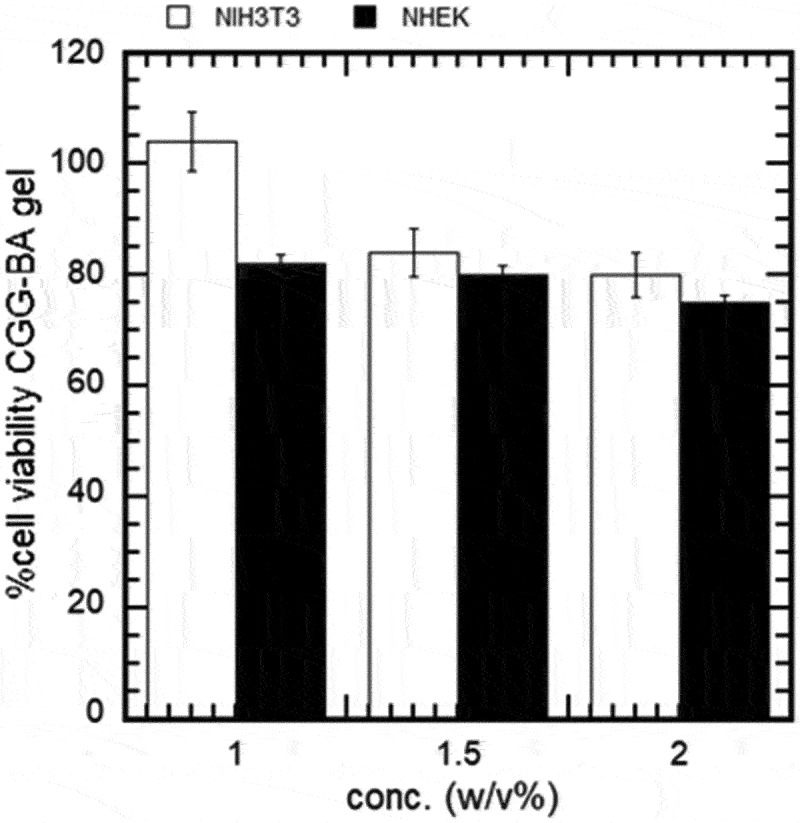
Cell viability test of CGG-BA hydrogels against NIH3T3 and NHEK cells.

The cytotoxicity was evaluated by incubating the hydrogels with NIH3T3 and NHEK cells for 48 h. CGG-BA hydrogels showed no toxicity to the cells within the examined concentration range and the results are shown in Figure 8. Images (Figure S8) of the cells were confirmed to be the same. The cell viability was about 80 ± 2% for NHEK cells, and the viability was between 80% and 110% for NIH3T3 cells with the hydrogel concentration of 1.0–2.0 w/v%. Although high concentrations of BA have been reported to show toxicity, combining the BA with CGG eliminated the effect [30]. Previous studies [19] suggested the safety of the CGG-based hydrogels for use as a biomaterial.

### 3.7. Ex vivo muco-adhesion: tackiness test, burst pressure test, lap shear test, and hydrogel degradation

**Figure 9.**
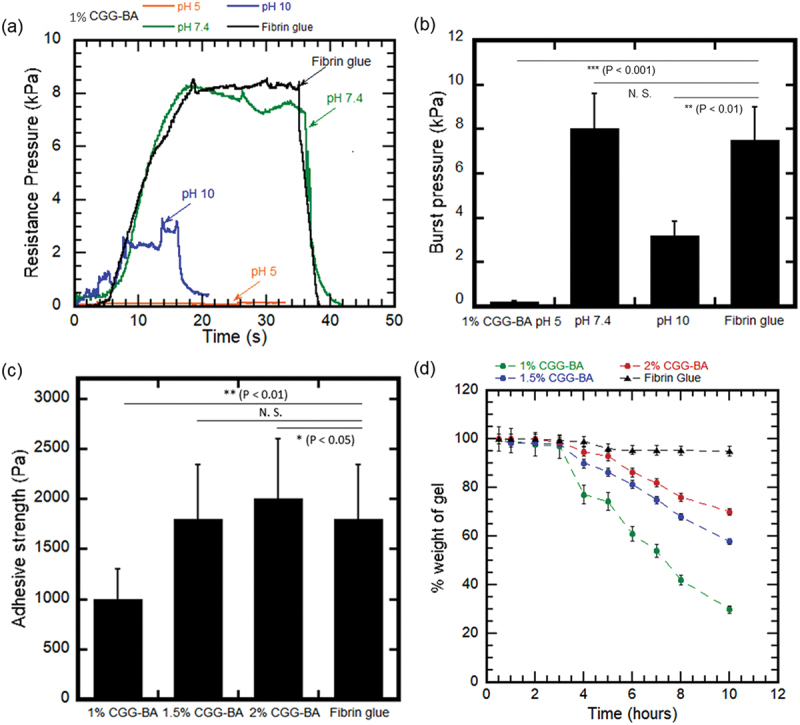
Results of *ex vivo* mucoadhesive test (a) Increase in resistance pressure with time on ejection of PBS through hydrogel attached to porcine esophageal mucosa, (b) Burst pressure of CGG-BA hydrogels at pH 5, 7.4 and 10, and that of fibrin glue control, (c) Adhesive strength of hydrogels calculated by lap shear measurement, (d) Degradation behavior of hydrogels applied on the mucosa during the incubation in PBS. (N. S. (non-significant), P < 0.05, P < 0.01 and P < 0.001 refers to the statistical significance).

The burst pressure of 1% w/v CGG-BA at pH 5, 7.4, and 10 are shown in Figure 9(a–d). At pH 5, the material exists as a solution and did not show any resistance to leakage of the solution, resulting in negligible burst pressure. At pH 7.4, the resistance to leakage increased to a maximum of 8 ± 2 kPa until the gel broke. This corresponds to the burst pressure of the gel. At pH 10, the burst pressure of the gel was 3 ± 1 kPa. Fibrin glue control measured a burst pressure of 7.5 ± 0.5 kPa. This value agrees with that of commercially available fibrin glue, previously reported [47], at similar experimental conditions. Thus, the burst pressure resistance of CGG-BA self-healable hydrogel (pH 7.4) was comparable to that of fibrin glue.

Both hydrogen bond and diol complexation would have contributed to the burst pressure resistance of the hydrogels. The results showed the same trends as that of tackiness test and yield stress measurement. As confirmed by the FTIR results, at pH 5, only hydrogen bonds existed, therefore the CGG-BA solution could not resist the fluid pressure. At pH 7.4, the bonding in CGG-BA hydrogels consisted of both hydrogen bonds and diol-complexation. At pH 10, the diol cross-linking within the hydrogel was responsible for the burst pressure resistance. However, the absence of hydrogen bonding at pH 10 resulted in lower burst pressure compared to pH 7.4.

The lap shear test was conducted to determine the adhesive force of the CGG-BA hydrogel on the mucosa (Figure 9(c)) [12,59,60]. The measured force could also include the cohesive force that holds the hydrogel together when pulled apart. We also used Fibrin glue as a control. At pH 5 and pH 10, the muco-adhesive force was not high enough to hold mucosa pieces together for measurement. At pH 7.4, 1%, 1.5%, and 2% w/v CGG-BA hydrogels showed an adhesive strength of 1 ± 0.4 kPa, 1.73 ± 0.6 kPa, and 2.8 ± 0.2 kPa, respectively. Fibrin glue control measured 1.5 ± 0.05 kPa, which was comparable to CGG-BA hydrogels. Both burst pressure test and lap shear test confirmed that CGG-BA hydrogels at pH 7.4 with self-healing ability have the potential to be used as a mucoadhesive. Statistical analysis was done using one-way ANOVA and Tukey’s Honest Significant Difference test.

The hydrogel degradation test in PBS showed that within 10 h, 1%, 1.5%, and 2% w/v CGG-BA hydrogel showed 72%, 40%, and 32% residual without dissolving and detaching from the mucosa (Figure 9(d)). About 95% of Fibrin glue remained after 10 h. The CGG-BA hydrogel was observed to adhere within a minute of contact to the mucosa. The retention of these hydrogels needs improvement to obtain a material durable enough to withstand physiological conditions [61]. As there are several stresses acting on the hydrogel in the GI tract, we will conduct future study to improve CGG-BA hydrogel retention to reach clinical standards [62]. In the future, the hydrogels potential for muco-adhesion in buccal cavity, nasal cavity, rectal lumen, etc., will also be considered.

Mucoadhesive polymer-based materials can be applied to sites with a mucosa lining, including conjunctiva of eyes, buccal, nasal, vaginal cavities, and gastrointestinal tract [1]. The interactions between mucoadhesive materials and mucosa include but not limited to interpenetration of polymer chains, adsorption via intermolecular interactions and wetting [5]. Among these, one of the important factors for muco-adhesion is the formation of hydrogen-bonds among the functional groups (hydroxyl, carboxyl, and amino groups) of the materials and mucosal lining at the application sites. Previous studies showed that polymers such as celluloses, poly(vinyl alcohol), and starch formed strong hydrogen bonds with mucosa [63,64]. In addition, the presence of other functional groups, such as carboxyl, ammonium, thiol groups, etc., contribute to muco-adhesion. Concentration of polymers also plays a role in muco-adhesion [15]. At very low concentrations, polymers do not interact sufficiently with the mucosa layer. An increase in concentration would lead to an increase in interpenetration between their chains. However, further increase would lead to inter-coiling of polymers that in turn results in prevention of interaction.

Yuan et al. analyzed the effect of the degree of substitution of cationic hydroxyethyl cellulose (CHEC) [15]. The study showed that as the degree of substitution increased, the hydrogel characteristics such as *G′* and *G″* values also increased, irrespective of polymer concentration. This was due to the high cross-linking degree caused by electrostatic interactions, hydrogen bonding, and entanglement of the polymer chains. Also, as the degree of substitution increased, the charge density increased, leading to an increase in adhesion energy. However, beyond a certain concentration the effect was not prominent. This is because, at very high concentrations (e.g. 33% w/v), the cohesive forces within the system increase, leading to a decrease in free polymer ends. Similar behavior is expected in the case of CGG, due to structural similarity between CHEC and CGG.

In this study, we fixed the mole ratio between CGG and BA. Interactions between hydroxy propyl guar (HPG) and BA, thoroughly analyzed by Wang et al. [52], showed that the mole ratio can bring about changes in the hydrogel characteristics. HPG-BA interactions occur via the interactions between hydroxyl groups on borate ion and cis-hydroxyl groups on HPG. There is the possibility of both intramolecular and intermolecular interactions. At pH values lower than 8, due to partial conversion of BA to borate ion, intermolecular complexes were formed rather than intramolecular complexes. This leads to the formation of low viscous samples at low pH. On comparison to HPG, the CGG structure consists of the quaternary ammonium group. Therefore, as the mole ratio of CGG to BA increases, adhesion is expected to increase, because an increase in cationic charge density would lead to possible electrostatic interactions with the sialic group in mucosa.

As evidenced by the *ex vivo* mucoadhesive tests, this study showed that CGG-BA self-healable hydrogel had the potential for use as a mucoadhesive. A schematic representation of the potential interactions between the hydrogel and mucin is shown in Figure S9. Possible interactions of CGG-BA hydrogels with mucin would include hydrogen bonding between the hydroxyl groups present in the hydrogel network with those of the oligosaccharides in mucin [65]. This interaction is pH responsive due to the conversion of boric acid into borate ion at higher pH. At low values of pH, for, e.g., at pH 5, boric acid would form weak hydrogen bonds with the oligosaccharides. As pH increases to neutral and alkaline range, dissociation of boric acid to borate ion occurs, leading to stronger hydrogen bonds with mucin. In addition, the cationic groups of CGG would interact with the carboxyl groups of sialic acid of mucin through electrostatic interaction [1,66]. Our experiments showed that CGG-based boronic acid hydrogels have the potential to be used as a mucoadhesive which can respond to the pH of the application site. Esophageal mucoadhesive strength of CGG-BA hydrogel was found to be comparable to that of fibrin glue. However, the type of tissue substrate used greatly affects the mucoadhesive potential. Mucosal drug delivery systems [13] (buccal, nasal, ocular, gastro, vaginal, and rectal) utilize the selective muco-adhesion and pH responsiveness of materials, making CGG-BA a suitable candidate.

Self-healing hydrogels have been previously reported to be used in applications requiring tolerance to high stretching conditions such as wound dressing on joints [67]. Due to the mechanical flexibility and resistance, these hydrogels are expected to replace the brittle gels used in clinical application [58]. This is due to their ability to reform after being subjected to physiological stresses such as bleeding and fluid leakage that occur in gastrointestinal conditions (Figure S10). It was evident from our study that self-healing hydrogels had better mucoadhesive potential compared with brittle hydrogels from the same CGG-BA precursor. In this respect, self-healing properties make them a promising biomaterial.

CGG is widely used in food [16], skincare [19,20], and hair products [18] due to their easy availability, reasonable cost, and biocompatibility. In addition, BA is a constituent of eye drops [68] used in clinics. Therefore, CGG-BA hydrogels have the potential for medical or health-care applications. The self-healing behavior of CGG-BA was pH dependent, thereby allowing external control over the healing process. It is to be noted that the durability of CGG-BA hydrogel needs to be improved. A trade-off exists between hydrogel toughness and rapid self-healing. Although such soft self-healable hydrogels still face challenges of stability within the dynamic and mechanically demanding environment of human tissues, they are promising for various biomedical applications.

## 4. Conclusions

We developed a new, quick gelling, pH responsive, self-healing hydrogel with the potential for mucoadhesive applications in the GI tract. CGG-BA showed a transition from a solution state at pH 5, to slimy hydrogel state at pH 7.4, to a brittle hydrogel at pH 10. Hydrogel formation occurred within 1 min, making it an easily preparable *in situ* cross-linkable hydrogel. The material did not show cytotoxicity, which was advantageous for biomedical applications. Furthermore, the self-healing property allows the hydrogel’s use for mucoadhesive applications in arduous clinical situations. This claim was supported by *ex vivo* mucoadhesive tests conducted to mimic physiological situations. The hydrogels’ mucoadhesive strength was comparable to that of fibrin glue. Hydrogen bonding within the system plays a crucial role in imparting both self-healing and muco-adhesion.

## Supplementary Material

Supplemental Material

Supplemental Material
