# Supplementary material for: pH responsive cationic guar gum-borate self-healing hydrogels for muco-adhesion
Source: Sci Technol Adv Mater. 2023 Feb 28;24(1):2175586. doi: 10.1080/14686996.2023.2175586 (PMC9990695; doi:10.1080/14686996.2023.2175586)
Supplement: Supplemental Material [file TSTA_A_2175586_SM2151.pdf]

Supplemental Information

pH sensitive cationic guar gum-borate self-healing hydrogels for muco-adhesion

Athira Sreedevi Madhavikutty[a], Arvind K. Singh Chandel[b], Ching-Cheng Tsai[c], Natsuko Inagaki[b], Seiichi Ohta[a,c] and Taichi Ito[a,b,c,*]

[a] *Department of Chemical System Engineering, The University of Tokyo, Tokyo 113-8656, Japan;*
[b] *Center for Disease Biology and Integrative Medicine, The University of Tokyo, Tokyo 113-0033, Japan;*
[c] *Department of Bioengineering, The University of Tokyo, Tokyo 113-8656, Japan.*

*Corresponding author
Taichi Ito, Ph.D.

Centre for Disease Biology and Integrative Medicine Faculty of Medicine,
Department of Chemical System Engineering, School of Engineering,
Department of Bioengineering, School of Engineering,
The University of Tokyo
Address: 7-3-1 Hongo, Bunkyo-ku, Tokyo 113-0033, JAPAN
E-mail: taichi@m.u-tokyo.ac.jp

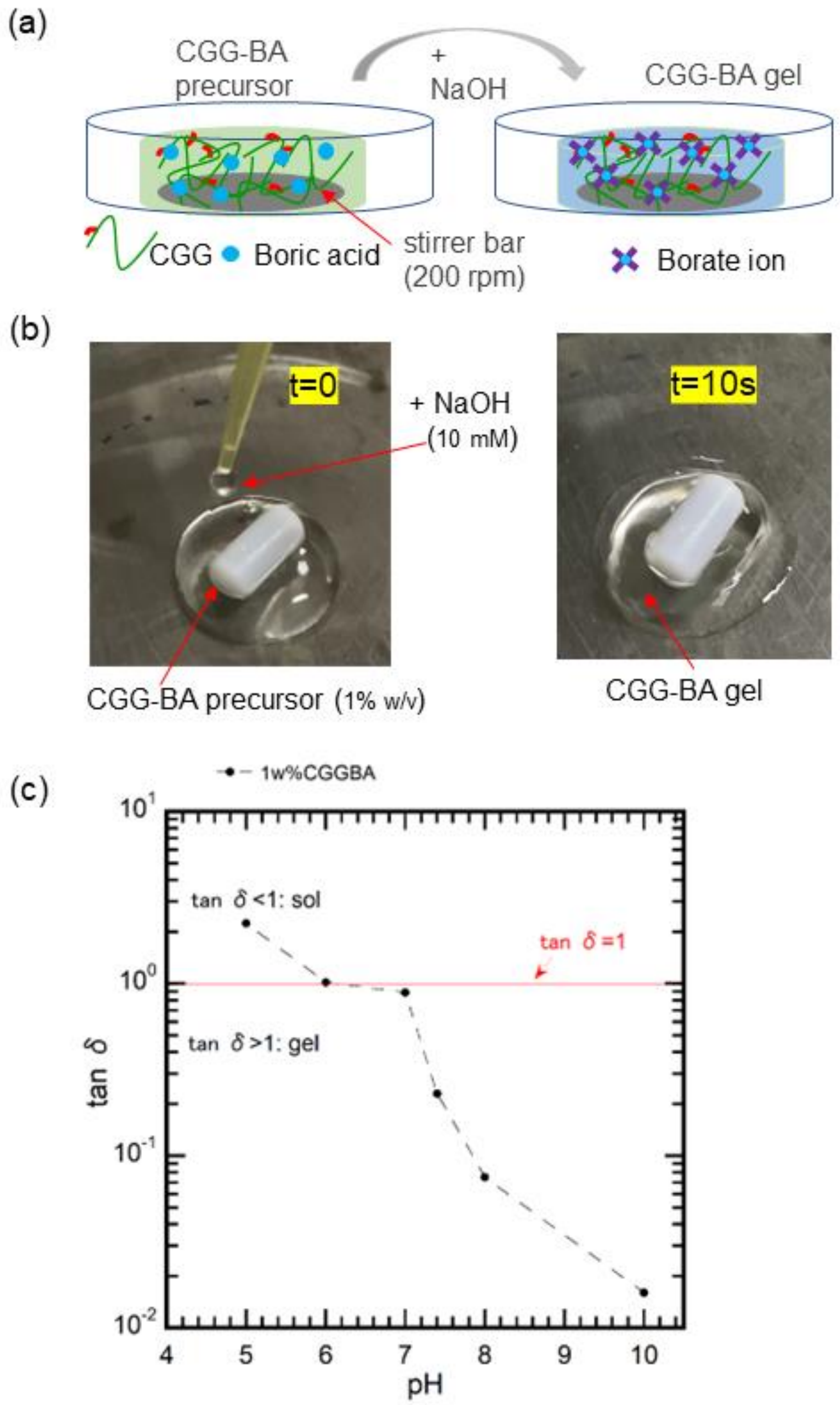

Figure S1. (a) Schematic illustration of gelation time determination, (b) Representative image of gelation time determination of 1% CGG-BA gel, (c) Effect of pH on loss factor of 1%w/v CGG-BA.

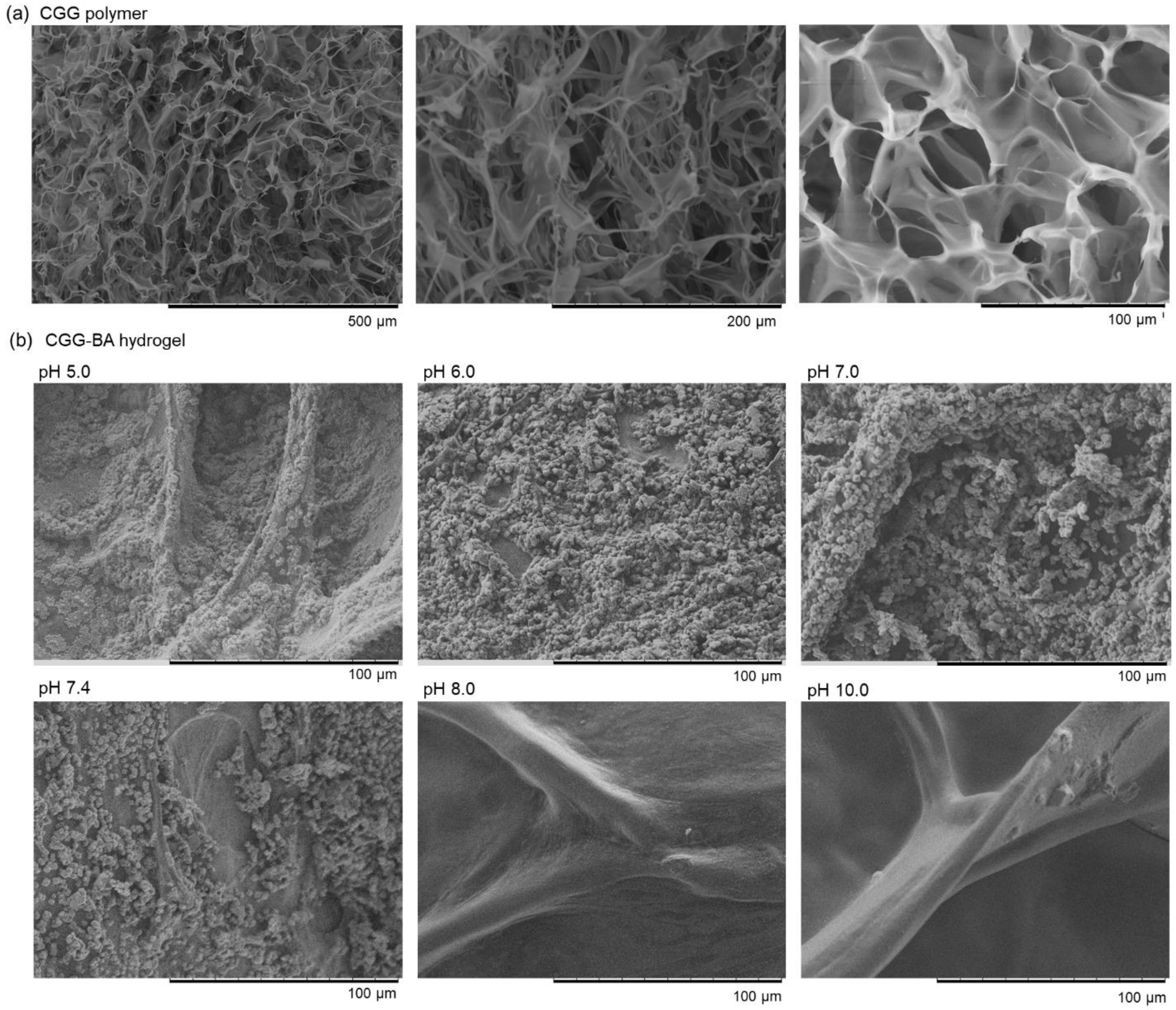

Figure S2. SEM images of (a) CGG polymer (resolution 500, 200 and 100µm), (b) 1% w/v CGG-BA at pH 5, 6, 7, 7.4, 8 and 10 with higher magnification than Figure 4.

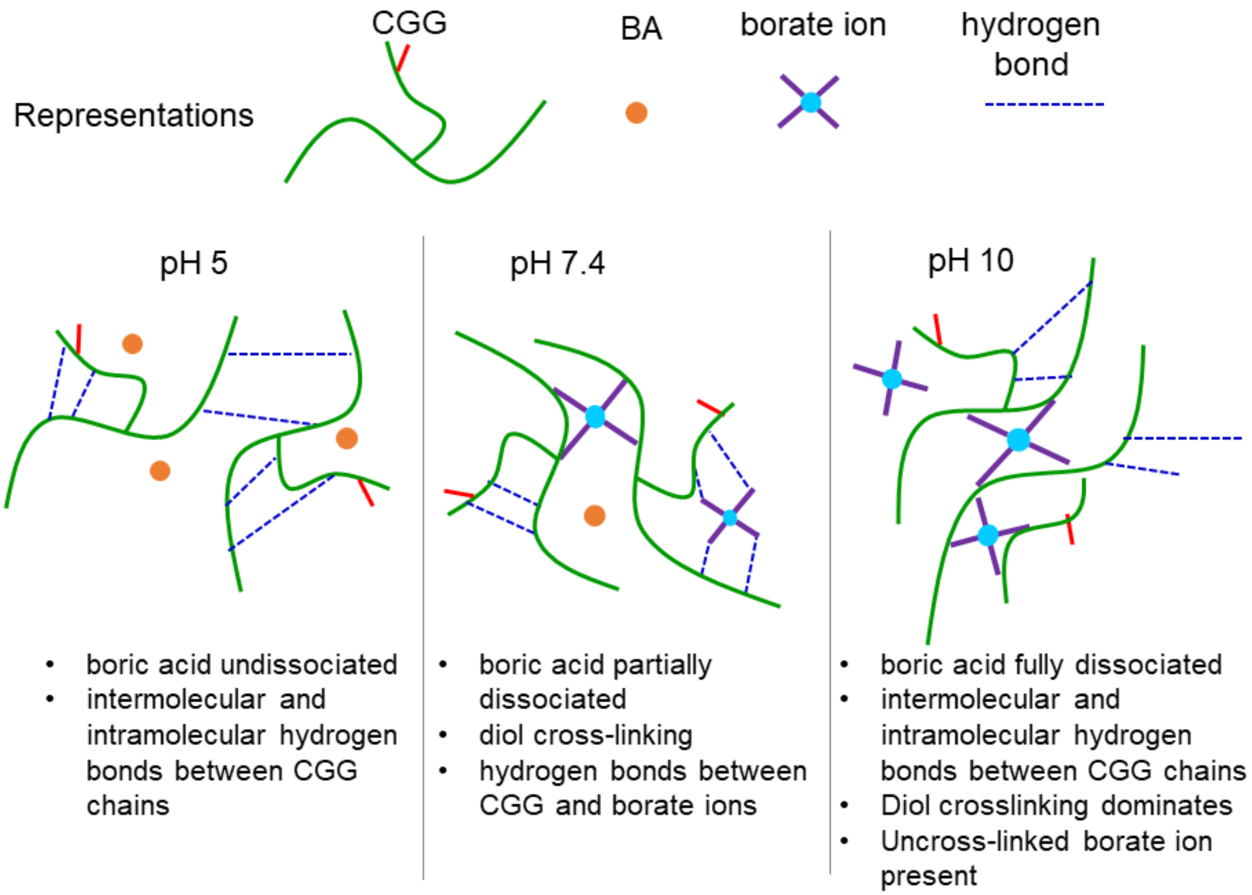

Figure S3. Schematic representation of cross-linking within the CGG-BA network at pH 5, 7.4 and 10.

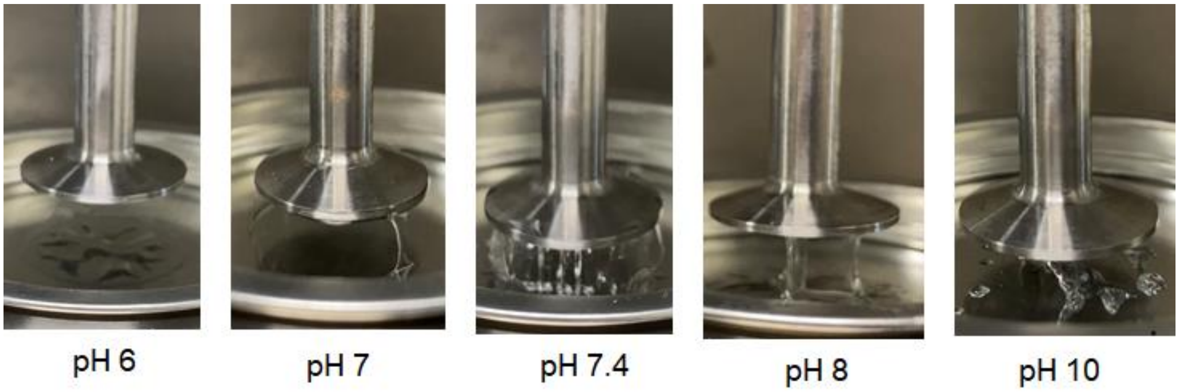

Figure S4. Appearance of 1%w/v CGG-BA with different pH during the tackiness test using rheometer

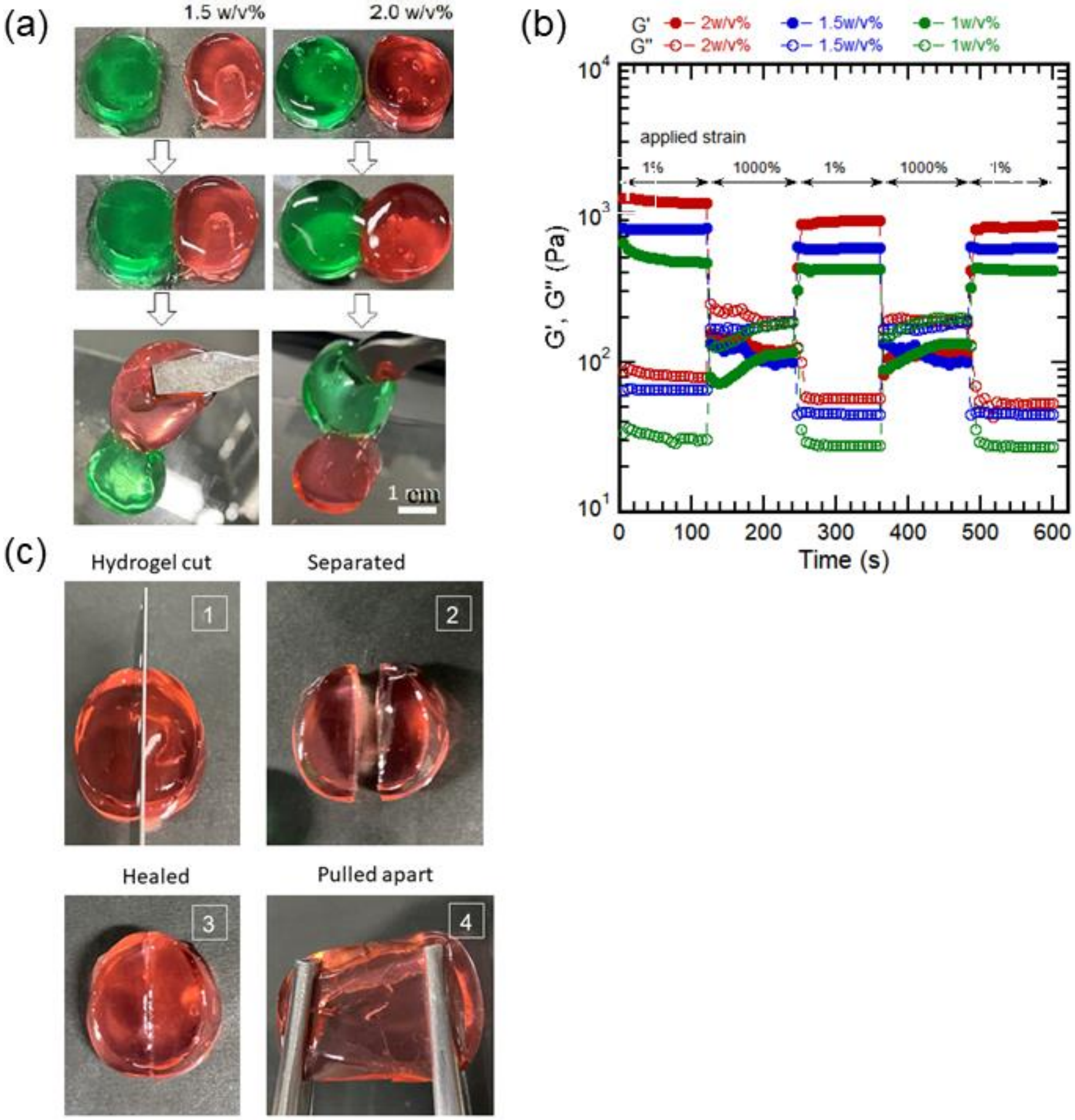

Figure S5 (a). Images of self-healing behavior of 1.5%w/v and 2%w/v CGG-BA hydrogel at pH 7.4. (b) Restructuring of CGG-BA hydrogels at pH 7.4, evaluated by the alternate amplitude sweep. (c) Image of 2%w/v hydrogel during the self-healing test. Hydrogel was 1: cut, 2: separated, 3: self-healed on contact, and then 4: withstood stress on being pulled.

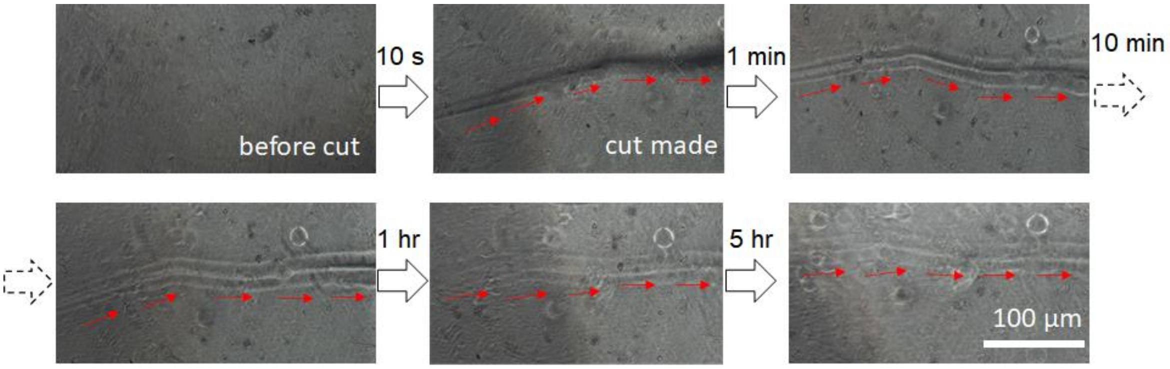

Figure S6. Microscopic visualization of self-healing by observing a cut made on the 1 % w/v CGG-BA hydrogel with pH 7.4. Red arrows are added as visual guide to observe the cut and its healing

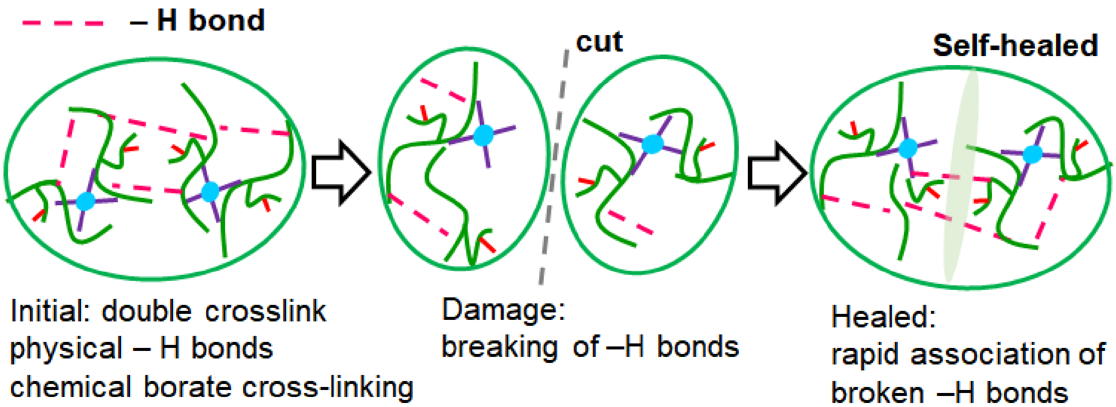

Figure S7. Proposed mechanism of self-healing within the CGG-BA hydrogel

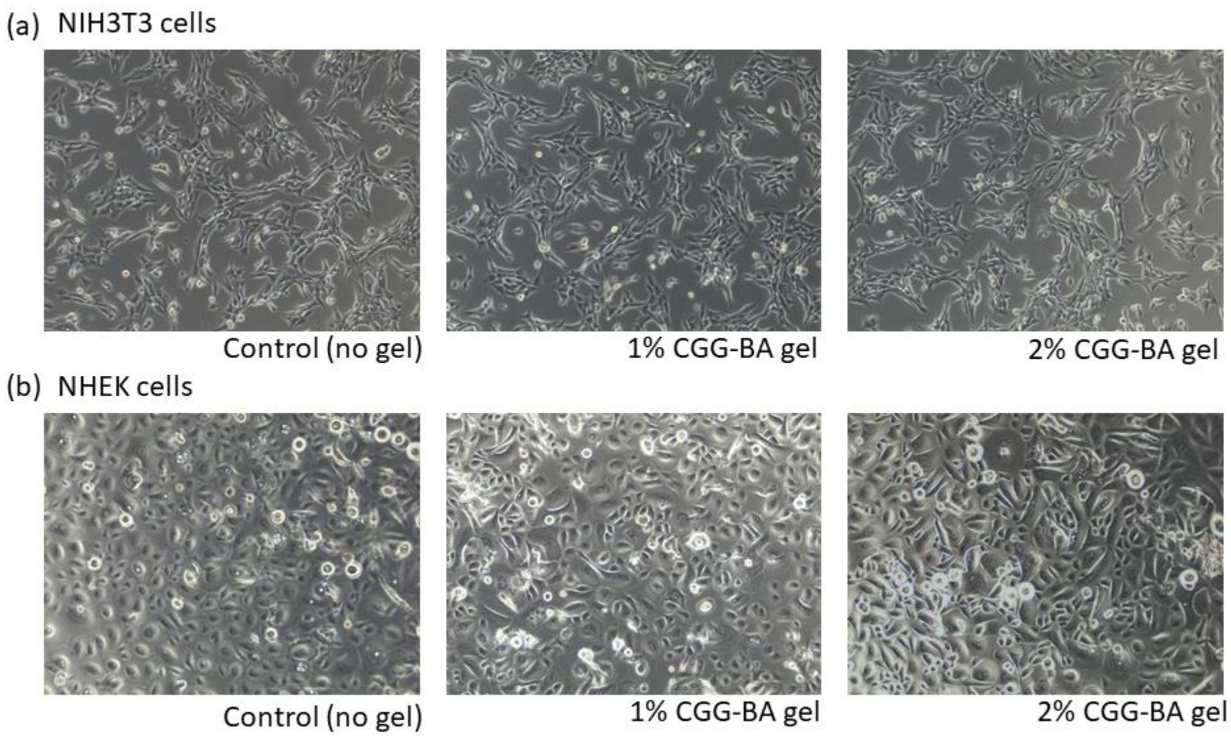

Figure S8. Optical microscopic images of (a) NIH3T3 and (b) NHEK cells after incubation with 1% or 2% CGG-BA hydrogels for 48 h. Cells without material application are also shown for comparison

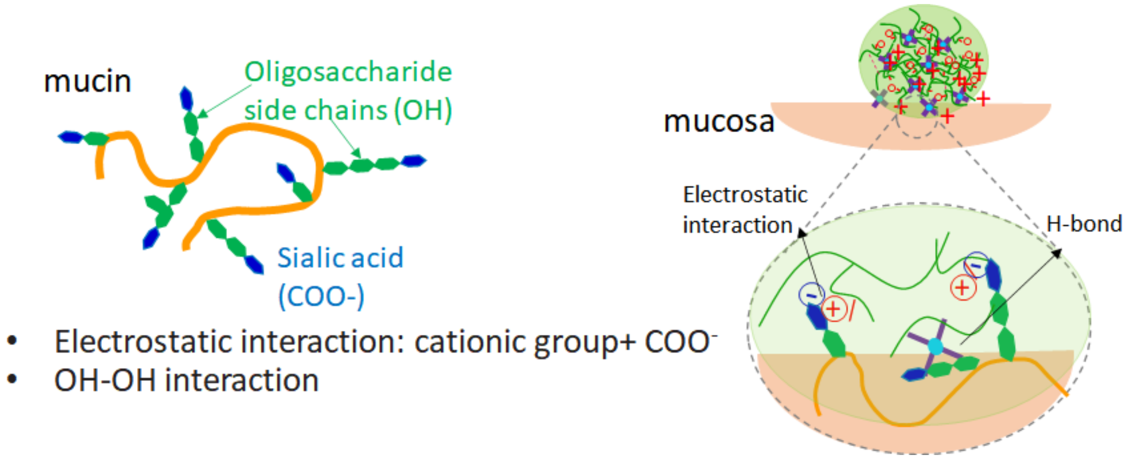

Figure S9. Possible interactions between mucosa and CGG-BA hydrogel

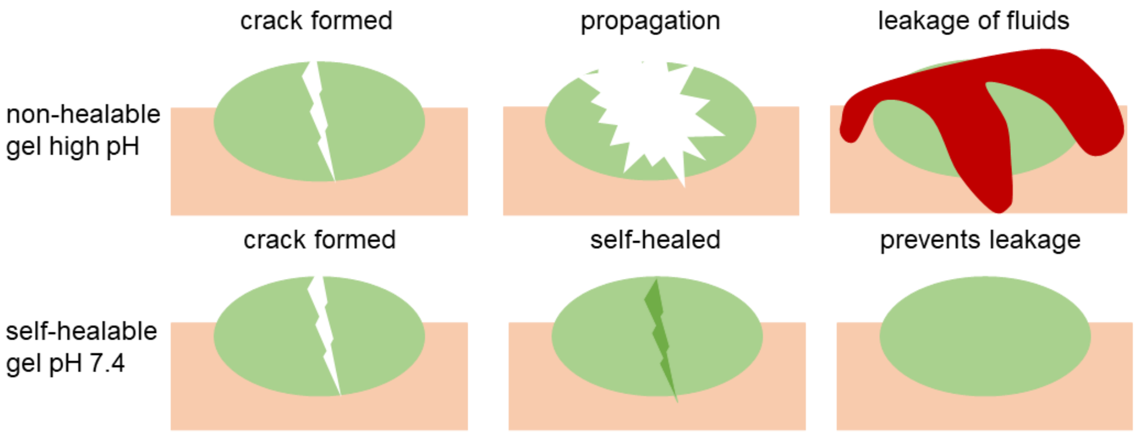

Figure S10. Schematic representation of the effect of self-healing on mucoadhesive behavior of hydrogels
